# Evaluation of the Impact of Different Types of Health Education on the Adoption and Preservation of Prohealth Attitudes in Preventing Cancer in Juveniles Younger than 18 Years

**DOI:** 10.1007/s13187-014-0730-y

**Published:** 2014-10-02

**Authors:** Krzysztof Adamowicz, Marta Zalewska, Mikołaj Majkowicz, Jan Maciej Zaucha

**Affiliations:** 1 Regional Oncology Centre in Gdańsk, Gdańsk, Pomorskie Poland; 2 Department of Prevention of Environmental Hazards and Allergology, Medical University of Warsaw, Warszawa, Poland; 3 Department for Research on Quality of Life, Medical University of Gdańsk, Gdańsk, Poland; 4 Department of Propaedeutic Oncology, Medical University of Gdańsk, Gdańsk, Poland; 5 Zakład Propedeutyki Onkologii, Gdańskiego Uniwersytetu Medycznego, Powstania Styczniowego 1, 81-519 Gdynia, Poland

**Keywords:** Cancer, Knowledge, Lifestyle, Juveniles

## Abstract

Reduction in the incidence of cancer can be achieved through appropriate health behaviors. We hypothesized that education would improve knowledge of cancer prevention, and this, in turn, will affect and individual’s readiness to modify lifestyle. The aim of this study was to assess the impact of cancer prevention education on adopting and preserving prohealth attitudes among high school students in Poland. Research participants were 307 high school students varying by gender, place of residence, parents’ education, and type of school education. Participants were divided into five groups, of which four were educated using different methods according to classification methods based on the concept of multilateral learning. The fifth (control) group was not educated. The effects of education were assessed 1 month and 1 year after education. General knowledge about cancer and healthy lifestyle level before education was low. After education, both increased compared with the control group. There was a clear relationship between level of knowledge and readiness to adopt and healthy attitudes and behavior. The most effective method of education was a discussion and a lecture by means of teaching complex. Education significantly improved generally low knowledge about cancer and healthy lifestyle in high school students. This indicates the urgent need to implement such educational programs.

## Introduction

Healthy lifestyle is defined as all kinds of human activities that maintain, strengthen, and restore health. Health education is a complex process during which students are taught to care for their own health and the health of society. Its main objective is to propagate knowledge about health itself, provide counselling in relation to health-related risks, and promote a healthy lifestyle. Health promotion means raising individual and societal awareness concerning health and factors affecting health, as well as developing and strengthening health resources of the individual and the community. In Poland, there are many alarming tendencies to neglect a healthy lifestyle.[1]. The exact characteristics of attitudes toward smoking among school children was presented in a nationwide study done in the framework of the Global Youth Tobacco Survey [2, 3] Results showed that 64 % of boys and 53 % of girls aged 13–15 years have smoked cigarettes; 30 % of boys and 21 % of girls have tried smoking before the age of 10 years. Furthermore, 25 % of boys and 21 % of girls admitted to smoking in the previous month. This figure is approximate to those presented in other countries of the European Union. [4] The diet of Polish adolescents was observed to be excessively high in flour products, sweets, and animal fats and too low in fish, vegetables, fruit, and products containing probiotics. [5] The Health Behaviour in School-aged Children (HBSC) study revealed a number of irregularities in the field of health behaviors among Polish youth. In 2010, 76 % of Polish youth aged 11–15 years had tried alcoholic beverages. Low physical activity during leisure is reported by almost every third child. Another worry is the teenager’s eating habits: 60–70 % of youth eat no vegetables or dark bread, 41 % have no milk, and 41 % candy and sweet drinks daily. Every sixth teenager goes to school without breakfast, and one in five do not eat a second breakfast at school. [6]

After heart and cardiovascular diseases, cancer constitutes the second leading cause of death in developed countries. There are two main groups of factors related to the development of cancer: environmental and genetic. The most important are environmental factors, including lifestyle and diet. It is estimated that ~70 % of malignant tumors are a consequence of harmful environmental factors.[7] While it is not possible to change genetic determinants, it is possible to eliminate the adverse factors related to lifestyle by directing people’s thinking toward a healthy lifestyle. To achieve the highest possible efficiency of such targeted actions, it is necessary that individuals acquire appropriate knowledge, which can be obtained by an adequate educational process. Therefore, health education and promotion play a fundamental role in promoting and implementing a healthy lifestyle.

The ultimate effect of health education should be reduction of overall morbidity. Assuming that factors related to lifestyle are the cause of up to 70 % of cancers, it can be concluded that the main goal of health education in cancer prevention should be to promote a healthy lifestyle. The effectiveness of such education could be measured by the percentage of people changing or expressing a readiness to change their lifestyle.

The main source of education is school, although the family home continues to influence the development of knowledge, as well as good and bad habits. In the preamble to the curriculum for schools is the sentence: “An important task of the school is also health education, carried out by the teachers of many subjects, which aims at developing students’ skills of caring for their own health and the health of other people”.[8] This particularly significant statement makes health education of vital importance among the tasks of the contemporary school. According to the widely accepted approach, health education should mean the conscious creation of projected opportunities for learning and facilitating behavioral changes. According to a report of the Department of Health Promotion in Cracow, juveniles themselves considered the following elements as the most important topics in health education: preparation for sexual activity, nutrition, physical activity, drug addiction, mental hygiene, disease prevention, and preparation for family life.[9] Such an array of broadly expressed needs can be a sign of the lack of knowledge in the field of health care, and we showed that knowledge about cancer among adolescents was low [10], which is part of a trend reported in similar studies.[11, 12]

The success of health education results from the use of certain topics, resources, methods of action, and structural forms. It seems that the best time to start health education is the age at which an individual can fully understand dangers resulting from the lack of health care and before health is permanently damaged. For this reason, adolescents seem to be the most appropriate age group within which health-promoting education should start. The prerequisite for effective acquisition of healthy habits by juveniles is the incorporation of health education into the educational process. All educated individuals should acquire knowledge about health and the skills to care for it. This consequently should lead to lifestyle changes. The aim of this study was to assess short- and long-term impact of health education on cancer prevention by adopting and preserving prohealth attitudes among students. We hypothesized that education would improve such knowledge and, in turn, positively affect teen’ readiness to modify their lifestyle.

## Materials and Methods

This study initially involved 307 second-year students of secondary schools with different profiles and varied by gender, place of residence, and type of parental and scholarly education. The protocol and consent form were approved by the Medical University of Gdańsk and review boards of all participating institutions. All participants provided informed consent (Table 1).

**Table 1:** Study group characteristics before education

Characteristic	Number (% of total)
Sex	
Boys	133 (43.6 %)
Girls	172 (56.4 %)
Place of residence	
City	186 (61 %)
Village	119 (39 %)
Parental education	
Father	
Lower than average	81 (26.56 %)
Average	115 (37.70 %)
Higher than average	109 (35.74 %)
Mother	
Lower than average	99 (32.46 %)
Average	100 (32.79 %)
Higher than average	106 (34.75 %)

The first phase of the study analyzed health-promoting behaviors of the juveniles assessed and evaluated their knowledge about cancer, the role of screening tests in early cancer detection, and about cancer prevention. Study participants were divided into groups according to systematic sampling, with the interval k = 5, which was based on results of the test on cancer knowledge (61 students in each group, comparable in terms of age, gender, and place of residence). In four of them, different teaching methods were employed, according to the classification of methods based on the concept of multilateral teaching.[13] Method 1 (assimilation of knowledge using simple methods): this involved conducting two 45-min lectures within one week and ending in a 15-min discussion. Method 2 (assimilation of knowledge by using complex methods): this involved conducting two 45-min lectures within a week, supported by audiovisual means of communication (PowerPoint presentation with tables, pictures, and short film). Method 3 (independent inquiry, learning by discovery) was a task in which the young people were asked to write a short note on cancer prevention, which was evaluated by a clinical oncologist and discussed in the entire group. Method 4 (valorization–learning by experiencing) was an interview with a patient suffering from a cancer that could have been prevented by lifestyle modification (lung cancer; early breast cancer; 45-min interview week after week). The fifth group (control group) was not educated.

In the second stage an educational program based on previously obtained results was created (model of a lecture lasting 70 min and covering: general knowledge about cancer, lifestyle impact on the development of cancer and knowledge about the possibilities of early detection of cancer (ratio resulted from the knowledge of young people surveyed in different areas in the questionnaire before education). General knowledge about cancer was presented: definition, types, oncogenesis stages, genetic predisposition, incidence and mortality statistics in Poland and in world, diagnostic and therapeutic options. The impact of lifestyle was discussed: the definition of a healthy lifestyle; the role of prevention, a healthy lifestyle, smoking, alcohol consumption, diet, sun exposure, sexual behavior, self-examination, possibility of early cancer detection, the role of the GP, and screening for and early detection of cervical, breast, colorectal, and early symptoms of other cancers. On this basis, a multimedia presentation was prepared.

In the next phase of the study, 2 and 12 months after the education, healthy behaviors and knowledge about cancer were assessed again, and results were compared with those obtained before education. This allowed assessing whether education improved knowledge about cancer, its prevention, and the adoption of lifestyle changes to promote healthy behaviors. We used a diagnostic survey consisting of two questionnaires: the first evaluated knowledge about cancer; the second was a questionnaire, Health Behaviour Inventory (HBI). This questionnaire determines the intensity of healthy behavior and lifestyle. Given the frequency of particular behaviors indicated by the participants, we established the intensity of behaviors conducive to health and the intensity of the four categories of healthy behaviors: healthy eating habits, disease-preventing behaviors, health practices, and positive mental attitude.[14]

Statistical analysis included descriptive statistics such as arithmetic mean, standard deviation (SD), and median. To test the study hypotheses, Student’s *t* test, chi-squared test, and analysis of variance (ANOVA) [15] were used, with the level of significance *p* < 0.05.

## Results

### Assessing Cancer-Related Knowledge

The mean raw point value regarding knowledge about cancer before education was 64.9, with a maximum of 136. Eight weeks after completion of the education program, the mean value increased statistically significantly to 88.25. A year after education completion, the mean value decreased to 80.49 but remained statistically significantly higher (*p* < 0.01) than before education.

**Fig. 1:**
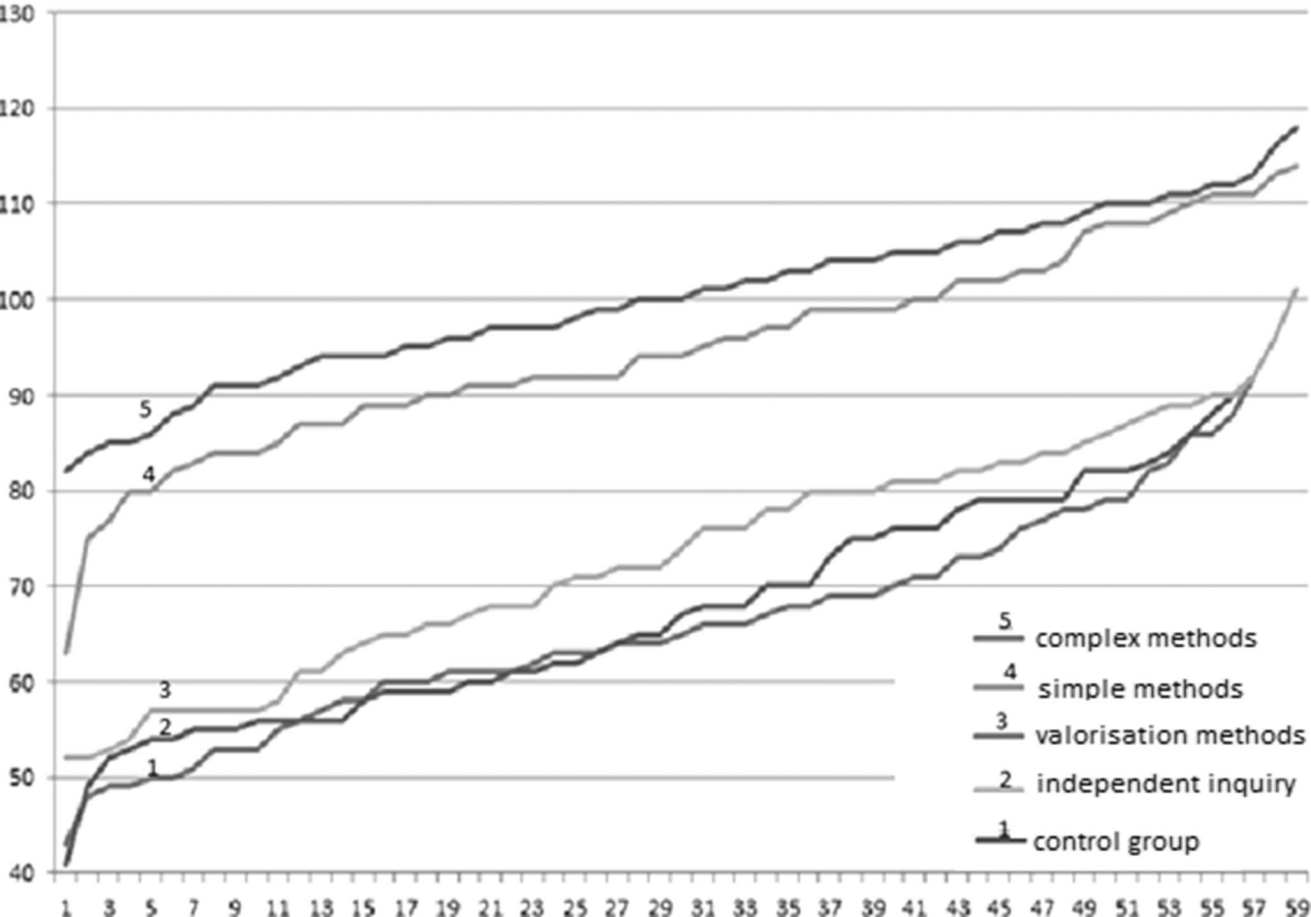
Growth in cancer knowledge in each group in relation to average results before education prograqm. Results are smallest to largest priorities. The most effective method of education was the complex method: line no 5

The 33 Student’s* t* tests performed on raw data before and 8 weeks after the education for response pairs (*n* = 244) demonstrated a statistically significant increase in the level of knowledge in all three thematic groups analyzed (*p* < 0.01). A year after the education, a re-examination (*n* = 234) demonstrated a statistically significant increase in the level of knowledge (*p* < 0.01). Eight weeks after education, each type of education brought a benefit, calculated as the average score at the survey after education, in relation to the score prior to education, and in relation to the control group (Table 2). A year after the education, not every kind of education brought a benefit, calculated as the average score at the survey after the education, compared with the score before education and to the control group. The biggest benefit was observed in the group educated using complex teaching means (method 2), the lowest in the group educated using the method of valorization (method 4) (Fig. 1). In the groups educated with methods 1 and 2, the effect of education remained after 1 year). Results obtained in method 2 were significantly better compared with all other groups (*p* < 0.05; methods 1, 3, 4), to the results before education (*p* < 0.01), and to the control group (*p* < 0.01). In the case of method 3, the increased level of knowledge assessed 2 months after education was lower compared with the groups educated by method 1 or method 2. More importantly, the effect of education that remained 1 year after education decreased by almost a half. The worst results were obtained using method 4, which 8 weeks after education obtained the lowest score of all groups (72.93), although overall results were higher than in the control group. In the noneducated control group, the level of knowledge did not change: 1-year later, results remained lower than in the other groups.

**Table 2:** Results of oncological knowledge before and after education provided by four different methods and compared with controls

Method	Knowledge of oncology				
	Before	2 months after	*P* value*	1 year after	*P* value*
1	64.43	95.46	<0.01	94.95	<0.01
2	64.67	103.95	<0.01	100.2	<0.01
3	64.88	80.69	<0.05	73.44	>0.05
4	65.14	72.93	>0.05	65.60	>0.05
No education	65.37	68.05	>0.05	67.35	>0.05

*Method 1* simple methods;* method 2* complex methods;* method 3* independent inquiry, learning by discovery;* method 4* valorization–learning by experiencing;* No education *control group

*Student’s* t* test

### Assessing Lifestyle

Initially HBI scores were low. In a multivariate analysis, we found a statistically significant difference in juvenile lifestyle between genders (*p* < 0.01):, with girls having the healthier. There was no relationship between a youths’ lifestyles and mothers’ (*p* = 0.55), fathers’ (*p* = 0.82), or combined parental (*p* = 0.72) education levels or place of residence (*p* = 0.96). Similar to results regarding knowledge about cancer, we observed a trend (*p* = 0.054) toward healthier lifestyle in the young people who attended the class with a biochemical profile component compared with other students. The most important finding was a statistically significant relationship between respondents’ lifestyle and knowledge about cancer (*p* < 0.01): those with knowledge had a healthier lifestyle.

Eight weeks after cessation of the education program, the mean value for each question concerning healthy behavior was statistically significantly higher than before the education. The 24 Student’s* t* tests for pairs of responses in four ranges performed on raw data before and after the education program showed an increase in the level of knowledge in all four analyzed areas.

After their education, female adolescents still led healthier lifestyles compared with male study participants (*p* < 0.01). However, no other earlier studied factors had statistically significant relationships with lifestyle, including the biochemical profile of the student class (*p* = 0.078). After the education program, the positive relationship between knowledge about cancer and a healthier lifestyle was observed in all categories of lifestyle. Each type of education brought a benefit, calculated as the average score at HBI after education, compared with the score before education and in relation to the control group. As was shown regarding increased knowledge in oncology, the biggest benefit was found in the group educated with complex educational means.

**Fig. 2:**
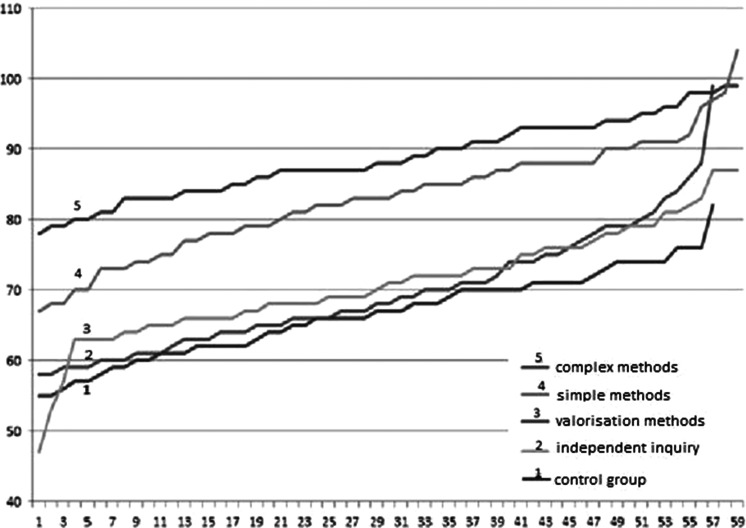
Increased results obtained in the Health Behavior Inventory of youth in each group in relation to average results before education. The most effective method was the complex  method: line no 5

A year after education, the 24 Student’s* t* tests performed on raw data before and after education for pairs of responses in four ranges still showed an increase in knowledge levels in all the four analysed areas of the general HBI. Again, the healthiest lifestyle was found among female adolescents (*p* < 0.01), and no statistically significantly better lifestyle score was obtained by respondents attending the class with a biochemical profile (*p* = 0.076). The correlation between lifestyle score and knowledge of cancer remained statistically significant (*p* < 0.01). However, those with higher knowledge about cancer had a healthier lifestyle. The biggest benefit was found in the group educated using complex means (Fig. 2).

Each type of education provided a benefit, calculated as the average score in the HBI at 8 weeks after in relation to prior to education and to the control group (Table 3). One year after education, the statistically significant increase remained only in the methods 1 and 2 groups, with the greatest being in method 2 (+20.1). This was sustained 1 year after education (Table 3). Results in this group were significantly better than all other groups (*p* < 0.05) compared with before education (*p* < 0.01) and the control group (*p* < 0.01). In all categories, lifestyles improved. A statistically significant improvement was also documented in students educated with method 1 compared with methods 3 and 4 and to controls. All students achieved significantly better results 8 weeks and again 1 year after education (*p* < 0.01) compared with before education and the control group (*p* < 0.01). In all categories, lifestyles became healthier. Those educated with method 3 showed a statistically significant increase in knowledge 8 weeks after compared with before the education program (*p* < 0.01). However, the average score 1 year after education was not significantly different from before education and was significantly lower than methods 1 and 2. A similar trend was observed in the method 4 group: mean score 8 weeks after was significantly higher than before education and in the control group (*p* < 0.05),; the score 1 year after was similar to prior to education and was no different from the control group. In this group, lifestyle improved only in two categories: health practices and proper eating habits. In the control group HBI assessment did not change (Table 3).

**Table 3:** Health behaviors before and after four methods of education

Method	Index of health behavior				
	Before	2 months after	*P* value*	1 year after	*P* value*
1	71.6	84.2	<0.01	82.98	<0.01
2	70.8	90.9	<0.01	90.4	<0.01
3	69.2	82.9	<0.01	70.98	>0.05
4	69.9	78.6	<0.01	69.9	>0.05
without education	68.1	68.5	>0.05	66.56	>0.05

*Method 1* simple methods;* method 2* complex methods;* method 3* independent inquiry, learning by discovery;* method 4* learning by experiencing;* no education *control group

*Student’s* t* test

## Discussion

The central hypothesis of this study was the assumption that education will improve knowledge of cancer prevention and, this in turn, will affect readiness to modify lifestyle. Second, it was assumed that the optimal age for health education is when an individual can fully understand the dangers resulting from the lack of health care and whose health has not yet been permanently damaged. Therefore, secondary school youths were selected for the study as being in the formative period of their lives and being susceptible to lifestyle changes providing they consider them important.

Results showed that knowledge about cancer among young people was low. This suggests the insufficient role of the existing forms of education in this field being carried out in high schools. Nevertheless, it should be emphasized that knowledge about the influence of lifestyle on cancer morbidity and mortality was statistically significantly higher than knowledge of the basic concepts surrounding cancer and cancer screening programs. The reasons for this difference are unclear. It may be due to knowledge gleaned from parents, or more likely, the result of wide-ranging media attention in recent years providing information on and promoting healthy living, mostly financed by the European Union (EU). Discussion on the prohibition of smoking in public places in EU countries could also explain increasing awareness about the negative effects of smoking and its influence on the development of certain cancers [16]. A concern is that there seems to be a lack of knowledge of cancer prevention programs, which may be due to insufficient emphasis in schools on such knowledge. 

The concern surrounding lack of knowledge among adolescents about cancer-prevention programs is twofold: According to predictions, the current adolescent generation will have an increased cancer morbitity—and mortality—rate than their ancestors, making their participation in prevention programs highly desirable. Second, and perhaps the most important for practicing oncologists, increased knowledge could improve cancer detection among parents of educated youth. Most parents are at an age where breast cancer morbidity peaks and colorectal and cervical cancer are diagnosed. One would hope that educating students on cancer prevention and healthy lifestyle could become an effective way to inform parents, the potential recipients of prevention programs.

Our study confirm the validity of the assumed hypothesis concerning the relationship between cancer education and lifestyle. Low knowledge coexisted with low level of HBI. On the basis of these results, we can say that adolescents are underinformed about healthy lifestyle habits and behaviors, which may in part be a result of lack of education on the subject. As expected, female participants had a higher level of knowledge about cancer, including the impact of lifestyle on cancer morbidity. Healthier female lifestyles were also evident following cancer education.

The level of knowledge of young people evaluated immediately after education was much higher than before. In all survey questions, average scores were generally significantly higher than before education. The most important finding is the fact that after education, every answer about healthy behavior achieved statistically significantly higher scores than before education, resulting in a healthier lifestyle in each range. Moreover, education was the most important factor influencing HBI survey results, and this effect was maintained after 1 year. The effect of education was therefore permanent, and most importantly, so was the impact of education on creating healthy lifestyle.

Due to lack of standardized and verified methods of conducting health education, choosing the optimal method was not easy. One study objective was to determine which method was the most effective for increasing participant’s knowledge concerning cancer and lifestyle modification. Sustainability of lifestyle changes was positively influenced by method 2, consisting of lecture application of complex teaching methods. This is consistent with the conclusions drawn by Dale, described as “Dale’s Cone of Learning,” which shows the average ability to recall information depends upon the educational method used [17]. There is no doubt that the key to a successful presentation is to focus on the audience and their needs, not only on information considered to be important and correct. Teachers emphasize that to pass knowledge well, first, gain the recipient’s attention and interests. In times dominated by technology, it seems that the best possible way to achieve this objective is to involve the highest number of senses. If one needs to emphasize the most important ideas, information should be displayed longer. Thus, in a multimedia presentation, lectures are supported by a presentation with slides, photos, and/or a short video. It seems that the study reported here is the first evidence demonstrating the general rules of pedagogy in the field of health education. The next best way in terms of effectiveness was method 1, based on discussion and lecture using simple teaching means. This is consistent with the general trend in education and the contemporary processes of transition from paternalistic to partnership education.

Relatively good but short-lasting results were obtained using the independent investigation into knowledge. In view of results after 1 year, however, it seems that this form of education does not create a long-term impact, is not memorable, and certainly should not take the single form. Surprisingly, a weak though statistically significant result 8 weeks after education was obtained with the valorization method. One could, however, presume that this form of education will not increase proportionally all thematic areas of oncology and knowledge of all areas involved in a healthy lifestyle. That the effect was short lived confirms the hypothesis that the effect can be due only to fear of cancer.

To summarize, our primary goal was to identify any statistically significant relationship between the lifestyles of the surveyed youth and their knowledge about cancer. A healthier lifestyle was seen in young people who had higher knowledge of cancer. This proves the relationship between such knowledge and the readiness to adopt healthy attitudes and behaviors. In view of the general lack of knowledge about cancer among young people, as shown in this study, our work emphasizes the importance of developing prohealth education. It also provides an indication of which educational method to adopt. Any kind of education positively influence the every individual’s state of knowledge in the short term, but for the long term, only selected educational methods proved to be effective. The most effective method of education was assimilation of knowledge, i.e., discussion and lecture using complex teaching means.

Based on results of this study, intend to conduct a pilot youth educational program in schools in a district in the Province of Pomerania, Poland.

## References

[1] Szymborski J, Jakobik K (2008). The health of children and youth in Poland. Scientific editors. Wars.

[2] Przewoźniak K., Zatoński W. Smoking prevalence, patterns and determinants in Polish schoolchildren, Abstract Book and Programme of the 3rd European Conference on Tobacco or Health, Warsaw 2002

[3] Przewozniak K, Zatonski W, Global Youth Tobacco Survey Collaborating Group (2003). Differences in worldwide tobacco Use by gender: findings from the global youth tobacco survey. J Sch Health.

[4] Baska T, Sovinova H, Nemeth A, Przewozniak K, Warren CW, Kavcova E, Czech Republic, Hungary, Poland and Slovakia GYTS Collaborative Group (2006). Findings from the Global Youth Tobacco Survey (GYTS) in Czech Republic, Hungary, Poland and Slovakia—smoking initiation, prevalence of tobacco use and cessation. Soz Praventivmed.

[5] Wojtyła A, Biliński P, Bojar I (2011). Health behaviors among Polish adolescents in their own and parental opinion. Probl Hig Epidemiol.

[6] Currie C., Zanotti C., Morgan A., et al. Social determinants of health and well-being among young people. Health Behaviour in School-aged Children (HBSC) study: international report from the 2009/2010 survey Health Policy for Children and Adolescents, No. 6

[7] Pasz-Walczak G. Prevention. W Kordek R.(red) Onkology. Handbook for students and doctors. Via Medica 2007; 52–7

[8] Regulation of the Minister of Science and Higher Education of 12 July 2007 on standards of education for various fields and levels of education. (2007, Nr 164, poz. 1166).

[9] Miller M., Supranowicz P. Current problems of health education. The needs of the students concerning information about health and lifestyle. Problemy higieny 2002, 77.

[10] Assessment of the impact of cancer education on changes in adolescents lifestyle. Onkologia Polska; 2012; 15: 157165

[11] Public Opinion Research Center. Attitudes and opinions of young people about cancer. Warsaw 2007.

[12] Butwin A (2009). Knowledge about breast cancer prevention among students of secondary schools Gdansk.

[13] W.Okon. Basics of teaching. (red) W.Okon (in:) The didactic system., Warsaw 1972; 13.

[14] Juczynski Z (2011). Measurement tools in promotion and health psychology.

[15] R Development Core Team (2008). R: A language and environment for statistical computing.

[16] Tke anti-smoking law. Act of 8 April 2010 amending the Act on protection of health against the consequences of tobacco use and tobacco and the Act on the State Sanitary Inspection.

[17] Dale E (1959). Audio-visual methods of teaching.

